# Displaying Lipase B from *Candida antarctica* in *Pichia pastoris* Using the Yeast Surface Display Approach: Prospection of a New Anchor and Characterization of the Whole Cell Biocatalyst

**DOI:** 10.1371/journal.pone.0141454

**Published:** 2015-10-28

**Authors:** Marcelo Victor Holanda Moura, Giulia Pontes da Silva, Antônio Carlos de Oliveira Machado, Fernando Araripe Gonçalves Torres, Denise Maria Guimarães Freire, Rodrigo Volcan Almeida

**Affiliations:** 1 Instituto de Química, Universidade Federal do Rio de Janeiro, Rio de Janeiro, RJ, Brasil; 2 Departamento de Biologia Celular, Universidade de Brasília, Brasília, DF, Brasil; Weizmann Institute of Science, ISRAEL

## Abstract

Yeast Surface Display (YSD) is a strategy to anchor proteins on the yeast cell wall which has been employed to increase enzyme stability thus decreasing production costs. Lipase B from *Candida antarctica* (LipB) is one of the most studied enzymes in the context of industrial biotechnology. This study aimed to assess the biochemical features of this important biocatalyst when immobilized on the cell surface of the methylotrophic yeast *Pichia pastoris* using the YSD approach. For that purpose, two anchors were tested. The first (Flo9) was identified after a prospection of the *P*. *pastoris* genome being related to the family of flocculins similar to Flo1 but significantly smaller. The second is the Protein with Internal Repeats (Pir1) from *P*. *pastoris*. An immunolocalization assay showed that both anchor proteins were able to display the reporter protein EGFP in the yeast outer cell wall. LipB was expressed in *P*. *pastoris* fused either to Flo9 (FLOLIPB) or Pir1 (PIRLIPB). Both constructions showed hydrolytic activity towards tributyrin (>100 U/mg_dcw_ and >80 U/mg_dcw,_ respectively), optimal hydrolytic activity around 45°C and pH 7.0, higher thermostability at 45°C and stability in organic solvents when compared to a free lipase.

## Introduction

With the growing concern with cleaner, more direct and specific synthesis routes, biocatalysts are seen as an alternative to conventional processes using established chemical catalysts. These molecules of biological origin offer several advantages when compared to those used in traditional processes, such as less extreme reaction conditions, chemo, regio and stereoselectivity. Enzymes also tend to form less by-products and do not require complicated protection steps in order to achieve the final product [1,2].

As a result of the increase in the utilization of biocatalysts, the enzyme market grew considerably reaching USD 4 billion in 2012. It is predicted a 8.3% annual growth in this market reaching USD 7.2 to 8.5 billion by 2018 [3,4]. However, the extensive use of biocatalysts is hampered by the high cost of enzymes [5]. In order to overcome this bottleneck, several strategies to increase enzyme stability and decrease production costs have been employed. The Yeast Surface Display (YSD) is a strategy first developed in *Saccharomyces cerevisiae* in which a produced enzyme is attached to the outer side of yeast cell wall which acts as a support, not participating in the enzymatic reaction [6]. The enzyme is exposed through an immobilized protein used as an anchor, which effectively holds the enzyme to the cell. One of the advantages of YSD is that the biocatalyst undergoes little to no processing prior to its final application because production and immobilization of the protein occur in a single step [6–8]. It also avoids problems related to internalization of the substrate and enzyme recovery [9].

Proteins usually used as anchors include Flo1 and α-agglutinin from *S*. *cerevisiae* [6,10]. The anchor protein can make use of a glycophosphatidylinositol (GPI) domain to attach itself to the cell surface. The covalent binding of the anchor to the cell wall is strong and resilient. Alternatively, amino acid repeats found throughout the primary structure of highly hydrophobic proteins may also be used to interact with the cell wall thus acting as anchors [8,11].

In order to produce biocatalysts using display technologies, well-known microorganisms such as *Escherichia coli* and the yeasts *S*. *cerevisiae* and *Pichia pastoris* are often used as hosts [9,12]. However, there are many factors that affect the performance of the biocatalyst, such as promoter strength, anchor type, size of the anchor, host secretion system, enzyme size, conformation and availability of the active site. So, finding a balance between these aspects is no easy task [7,9,13]. The conformation of the enzyme and its interaction with the anchor and the surface of the host are very important in the activity of the biocatalyst [14,15]. Interactions with the active site of the protein also tend to modify catalysis, as shown by Sun *et al*. [14]. These authors showed that when the anchor protein was closer to the active site of the catalyst, a greater steric effect was observed and this theoretically affected the active site binding with the substrate.

The strategy of using not GPI anchors, but the interactions between ser/thr and the cell wall for the display of enzymes, in particular lipases, brings some advantages regarding catalytic activity [15]. As many lipases have their active site near N-terminus, proteins that have internal, repetitive motifs, such as Protein with Internal Repeats 1 (pir1) theoretically may be able to improve the performance of the catalyst by displaying the enzyme anchored in the C-terminus without hindering the catalytic site [6,16]. The search for new anchors that can display enzymes with less collateral effects to the cell and to the enzyme can bring advantages to the YSD technique.

Based on this strategy, the protein Flo1p form *S*. *cerevisiae* was used as bait in order to select an anchor candidate with the presence of an elevated number of regions with high serine/thr content. The Pir1 protein was also used to anchor lipases due to its Internal Repeats to study the expression of CALB anchored to the cell wall.

Lipases (EC 3.1.1.3) are currently described as triacylglycerol ester hydrolases. In aqueous media, they are capable of catalyzing the hydrolysis of ester bonds to form alcohols and organic acids. However, the presence of free water is an important factor to determine which reaction is to be catalyzed. If the nucleophile present in the medium is replaced, other reactions can be catalyzed such as esterification, transesterification and aminolysis [17,18]. Lipases may be applied in the most various types of industries, historically being one of the most important groups of biocatalysts for biotechnological applications [19,20]. Among this group of enzymes, lipase B from *Candida antarctica* (LipB) represents one of the most important biocatalyst described in the literature [21,22]. Despite of its industrial relevance LipB is still quite expensive, especially when considering the production of low value products (e.g biodiesel) [6, 23].

Aiming at the investigation of new anchors to harbor lipases and the reduction of cost for lipase production in this work, we describe the production of LipB displayed on the surface of *P*. *pastoris* using the YSD approach and the characterization of the whole-cell catalysts (WCC) produced. LipB was displayed using two different anchors: Flo9, a new anchor prospected in the genome of *P*. *pastoris* cell; and the Protein with Internal Repeats 1 from *P*. *pastoris* (Pir1), which has already been used to display the reporter protein GFP and the human α-1-antitrypsin [16]. Finally, we sought to characterize the biocatalysts produced in regard of optima temperature and pH, stability towards temperature and organic solvents.

## Methodology

### Bioinformatic analysis

For the exploration of new anchors in the genome of *P*. *pastoris*, alignments were made using the BLASTp tool found at the NCBI portal. The internal repeats found in Flo1 (aminoacid residue 278 to 1087) from *S*. *cerevisiae* were used a template for data mining. The sequences of membrane proteins, EGFP and the prospected lipases were obtained from the UniProt database. TMHMM 2.0, from the Center for Biological Sequence Analysis, was used for cell topology prediction to analyze the chimeras *in silico*.

### DNA manipulations

All DNA manipulations were carried out according to Sambrook *et al*. [24]. DNA extraction and band purification was performed with kits from Qiagen according to manufacturer’s instructions. Restriction enzymes and T4 DNA ligase were from New England Biolabs. Plasmid DNA was used to transform *E*. *coli* DH5α using the electroporation method.

### Gene synthesis and plasmid construction

Synthetic DNA constructs were synthesized by Epoch Biosciences (USA). The *P*. *pastoris PIR1* gene (with its native secretion sequences)[16] was synthesized in-frame with the *C*. *antarctica* lipase B (LipB) and cloned into the constitutive expression vector pGAPZB (Invitrogen), containing the glyceraldehyde- 3 phosphate dehydrogenase (GAP) promoter to form pGAPZPIRLIPB. Likewise, the reporter *egfp* (enhance green fluorescent protein) gene was fused to the putative *P*. *pastoris* anchor *FLO9* (Genome Net code F2QQL9-PICP7) and cloned into pGAPZαB (Invitrogen) to form pGAPZαFLO9GFP. Since it was unknown if *FLO9* coded for a signal peptide it’s entire coding sequence was cloned in-frame with the *S*. *cerevisiae* α-factor secretion sequence present in pGAPZαB. Both plasmids were double-digested with *No*tI/*Xba*I and their released inserts were swapped. Transformants were plated in selective LB Low Salt medium containing 25 µg/mL zeocin. The resulting plasmids were named pGAPZPIRGFP and pGAPZαFLO9LIPB. All 4 plasmids (pGAPZPIRGFP, pGAPZαFLO9GFP, pGAPZPIRLIPB and pGAPZαFLO9LIPB) were linearized with *Avr*II (a site present with the *GAP* promoter) prior to transformation of *P*. *pastoris* X-33 (Invitrogen) cells via electroporation. Transformants were selected on YPD plates containing 100 μg/mL zeocin.

### Screening of transformants

Transformants containing LipB were tested for lipase activity after plating on YPD medium containing tributyrin. Selected transformants were cultured in 96 deep well plates in liquid YPD medium containing zeocin 100, 200 and 500 μg/mL. The clones able to grow in 500 μg/mL of antibiotic were tested for lipase activity, measured using MUF heptanoate as the substrate according to Prim *et al*. [25]. Transformants containing GFP were selected by using a Typhoon fluorescence scanner 9000 and then cultured in 250 mL Erlenmeyer flasks and quantified by GFP fluorescence in a Cary Eclipse fluorimeter 490 nm.

### Expression in shake flasks

Cultivation of the transformants was performed in YPD medium and a minimum medium described in Maurer *et al*. [26], at 30°C (250 rpm and). Expression was monitored and O.D and activity were measured once every 24hours for 96 hours.

### Immunolocalization

Cells were collected by centrifugation and washed 5X with PBS buffer. The cell pellets were used with and without an incubation with *p*-formaldehyde for 1 h. The pellets were washed 5X with PBS buffer and blocked with 1% BSA. Cells were then incubated with Alexa 588 antibody anti GFP (1:20 dilution in PBS, 1% BSA) for 1h. After washing 5X with PBS buffer cells were analyzed in an Olympus Ax70 microscope with the Alexa 588 filter.

### Lipase activity

Twenty microliters of a known mass of suspension (25 mg/mL DCW) of extract were resuspended in sodium phosphate buffer 50 mM (pH 7.0). The activity test was carried out according to Prim *et al*. [25] using the 4-methylumbelliferyl heptanoate ester as substrate and the measurements were made on a Varian Cary Eclipse spectrofluorimeter. Standard enzymatic activity was determined at 45°C, where one unit of activity is defined as the amount of enzyme required to catalyze the formation of 1 μmol of MUF in 1 minute under the assay conditions.

Activity towards 5% (w/v) tributyrin was performed at 45°C by titrimetric activity according to Aguieiras *et al*. [27]. Lipase activity was determined by titration of the FFAs released by enzyme action after 20 minutes. One unit (U) of lipase activity was defined as the amount of enzyme that releases 1 μmole of fatty acids per minute under the assay conditions.

### Temperature and pH profile

To determine optimal temperature and activity of the WCCs, cell preparations were subjected to a Central Composite Rotational (CCR) factorial design type with three repetitions of the central point. The activity measurements were made using MUF-hep as the substrate and 50 mM sodium phosphate buffer. The model was generated with Statistica 8.0 software. Levels and coded variables are shown in Table 1.

**Table 1:** Levels and codified variables for the experimental design.

Variables		Codified Values			
	-1,41	-1	0	1	1,41
Temperature (°C)	30	34,4	45	55,6	60
pH	6	6,56	7	7,44	8

### Stability towards organic solvents

The biocatalysts had their initial activity measured at 45°C (100% activity). They were then washed 3X with MilliQ water and incubated in the selected solvent at 30°C. At determined times, aliquots were removed, washed 3X with MilliQ water and resuspended in the original activity conditions and the remaining activity was measured.

### Thermostability

For thermostability test, samples were incubated in 50 mM sodium phosphate buffer (pH 7.0) at different temperatures and at different times residual activity was measured under the initial conditions at 45°C (pH 7.0). CALB LIPOZYME soluble from Novozymes was used as control.

## Results and Discussion

### *In silico* prospection

Since most of the anchors used in YSD are derived from *S*. *cerevisiae* we performed an *in silico* prospection of the *P*. *pastoris* genome for a protein that could be used as a new anchor for whole cell catalysis in this yeast. In order to do this, the flocculin Flo1 from *S*. *cerevisiae* was used as a template for the search. Flo1 is a 1537 amino acids membrane protein bearing highly hydrophobic serine/threonine rich conserved domains. It has a GPI anchor domain which is usually not used for anchoring lipases. A possible explanation for this is due to the proximity of the C-terminus with the catalytic site of various lipases, such as *Rhizomucor miehei* lipase (Rml). Thus, it is the interaction of these conserved and repetitive hydrophobic domains from Flo1 that anchor the protein to the cell wall [11,28].

Initially, the prospection was made using the entire sequence from Flo1 as template. The main target found was Flo9, a protein from the family of flocculins, just as Flo1, but significantly smaller (536 aa). When aligned, Flo9 shows 24% identity with Flo1p. A more stringent search was made using another strategy. This time, as bait, the Flo1 repeated conserved regions, rich in serine and threonine (amino acid 278 to 1087) were used. Again, Flo9 showed a considerable identity with the bait.

The search criteria used in the present work differs from that used by Zhang *et al*.[10] who looked for GPI anchors and found 63 protein candidates. Khasa *et al*. [16] used amino acid repetitions as a criteria for prospection and identified two proteins from *P*. *pastoris* with high number of internal repeats, named Pir1 and 2 (Protein with Internal Repeats). For this reason, Pir1 was also used as anchor in the present work. As proteins containing GPI anchors also have many regions rich in serine and threonine [10,29] it is not surprising that some results of the two strategies overlap. This is the case of zonadhesin, a protein from the family of agglutinins that was used as an anchor by Zhang *et al*. [10] which was also identified in the present work.

In order to assess the cellular localization of the biocatalysts formed by fusing LipB with Flo9 and Pir1, cellular topology prediction analysis was made using several algorithms.These predictions of cellular topology take into account the content of serine and threonine, the putative domains and secretion signals present in the sequence. Flo9 showed a higher probability of being transmembranar than Pir1 as it showed a possible secretion signal in the N-terminal portion of the chimera. In the cell topology test, Flo9 scored higher than Pir1, a known transmembrane protein successfully used as an anchor, which suggests that it has the potential to be used as an anchor as well.

### Immunolocalization

**Fig 1:**
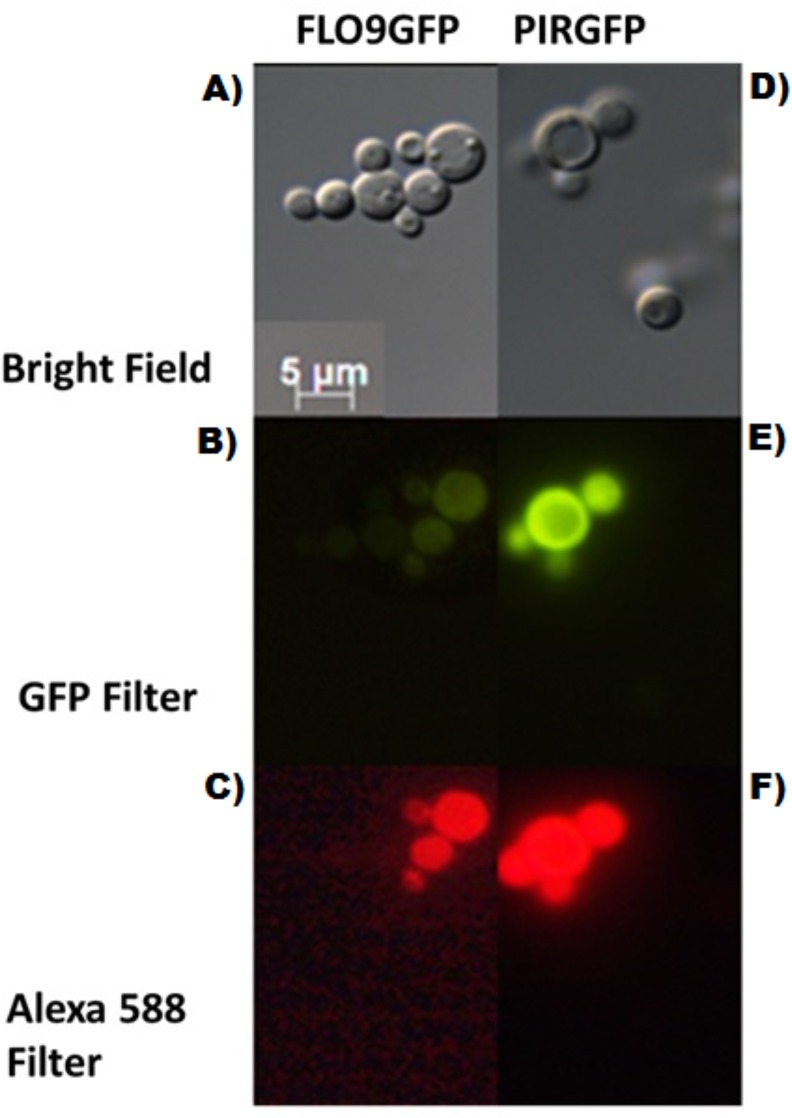
Fluorescence microscopy of *P*. *pastoris* cells producing FLO9GFP and PIRGFP. A) FLO9GFP—bright field B) FLO9GFP—wavelength of EGFP (485nm) C) FLO9GFP—wavelength of Alexa 588 (588nm). D) PIRGFP—bright field E) PIRGFP—wavelength of EGFP (485nm) F) PIRGFP—wavelength of Alexa 588 (588nm).

The protein anchors were fused to the reporter EGFP protein and immunolocalization was carried out (Fig 1). Imunofluorescence microscopy analysis was made to evaluate the correct addressing of the constructs and the successful anchoring role of the prospected proteins. Because the presence of EGFP fluorescence in the microscopy by itself does not guarantee the correct localization of the constructs in the cell wall an immunofluorescence assay was made using Alexa 588 anti-GFP antibody. This anti-body is GFP specific and the signal in the red microscopy could only be seen if there was a specific interaction between the protein exposed on the outside of the cell and anti-GFP antibody, thus showing the correct location of the construction. Similar assays have been described previously for the same purpose [8,30].The correct localization of the proteins are in accordance with the *in silico* predictions made using TMHMM and other cell topology prediction programs. Correct cell addressing may be a problem in expression and is a key feature for YSD, when the enzyme is immobilized in the cell wall.

### Expression of LipB fused to the anchors

**Fig 2:**
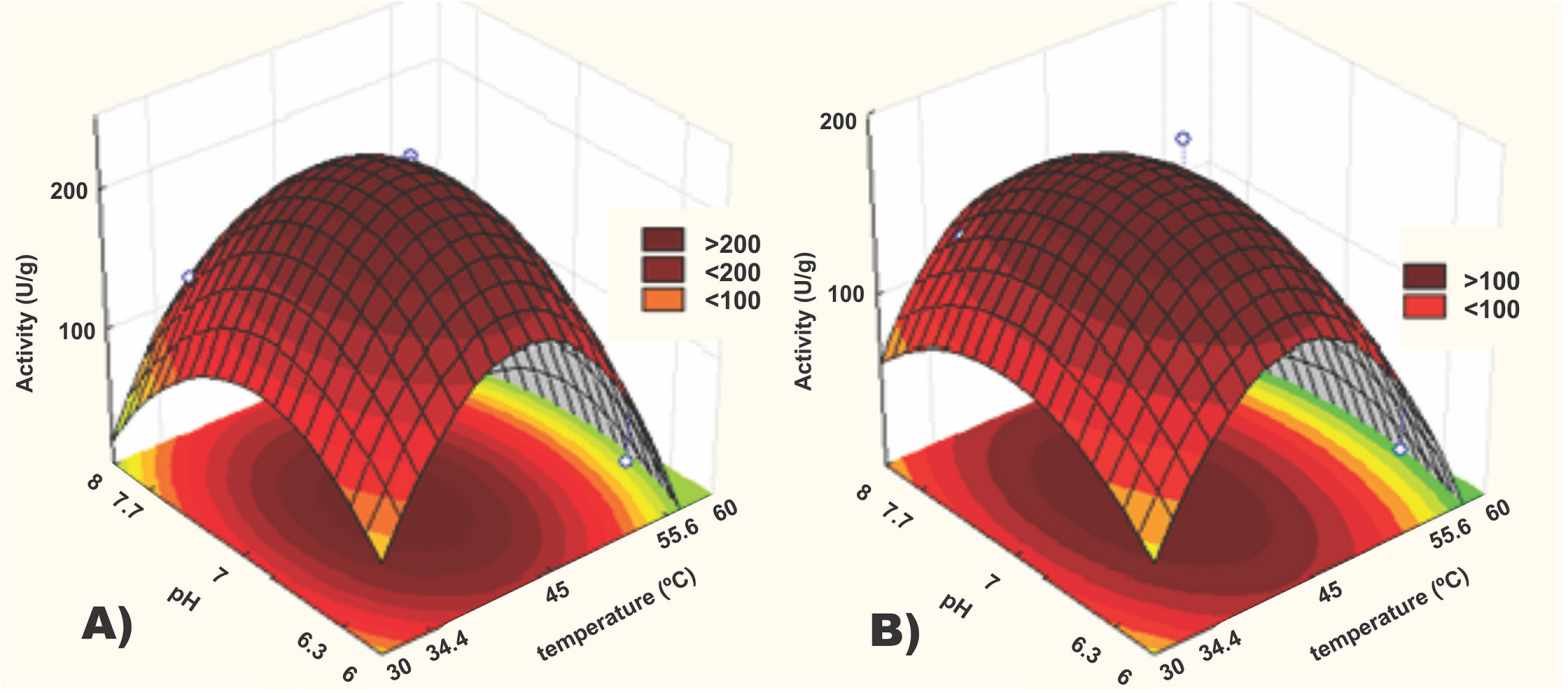
Response surfaces generated by Statistica 8.0 for the experimental design to determine optimum temperature and pH for (A) PIRLIPB and (B) FLO9LIPB.

We evaluated the use of Flo9 and Pir1 as anchors in a biotechnological applications using *P*. *pastoris* cells as WCC. For that purpose we determined some biochemical properties of yeast strains producing Pir and Flo9 fused to the LipB. We characterized these biocatalysts in terms of temperature and pH profile and stability towards solvents and temperature. Fig 2 shows the response surfaces generated from the experimental design made to determine pH and temperature profile. Temperature varied between 30°C and 60°C and the pH between 6,0 and 8,0.

Both constructions had similar biochemical characteristics, with higher activities in temperature and pH near 45°C and 7, respectively. However, the PIRLIPB construct showed specific activity approximately 17% higher than FLO9LIPB, with 226 U/L and 194 U/L, respectively. The equations describing the models adjusted for both enzymes are:

$$Activity\;of\;FLO9LIPB=176.97-38.21\times Temperature-64.78\times {Temperature}^{2}-23.51\times {pH}^{2}$$ (1)

R^2^ = 91.56%, F_calc_ = 25.3 F_tab_, 1.95

$$Activity\;of\;PIRLIPB=228.67-49.02\times Temperature-87.47\times {Temperature}^{2}-42.73\times {pH}^{2}$$ (2)

R^2^ = 91.25%, F_calc_ = 24.2 F_tab_ = 1.95

Both models had their F_cal_c>F_tab_, which indicates that lack of fit was not significate and the model could predict accurately the phenomenon described. The modeling showed that the interaction between the variables was not significate.

The temperature had both linear and quadratic influences in the models, which indicates that higher temperatures are better for activities, as expected, until it reaches a critical point. This can be explained as the catalysts suffering denaturation, and the effects turn to be detrimental to the catalysis. The pH variable had a mainly quadratic effect on the design, showing that when the pH turns too alkaline or too acid, the activity tends to decrease. The experiment also shows that beyond the optimum temperature, the catalysts tend to lose some activity, which also happens in more alkaline pH. Eom *et al*. [31] expressed LipB in *P*. *pastoris* and determined both the optimum temperature as well as optimal pH and 45°C and pH 7.0 for the free enzyme, which is consistent with the results obtained in the present work. Since the host used to express both constructs was the same, the heterologous expression, as expected, did not change the properties of the enzyme. However, the characteristics of the biocatalyst can be changed by immobilization. Sun *et al*. [14], immobilized CALB in macroporous resins in organic media, and found optimal hydrolysis temperature of 30°C and optimum pH of 6.0. The WCC developed in the present work did not exhibit that setback, maintaining the original characteristics of the enzyme. While comparing activities in different reaction conditions is a complicated task, the biocatalysts in the present work behaved similarly to others previously described [10,32,33]. Kato *et al*.[34] used the α-agglutinin anchor to express a mutated form of LipB. The catalyst showed hydrolytic activity towards *p*-nitrophenyl esters at 45°C and pH 7,0. Su *et al*. [33] expressed LipB fused to Sed1 and used the construct to hydrolyze *p*-nitrophenyl esters at 45°C and pH 8.0. In both cases the optimal temperature of the enzyme did not vary.

The optimal conditions found using the response surfaces were used to test a different substrate, tributyrin. Both catalysts showed activity towards the substrate. Again, PIRLIPB with slightly more active than FLO9LIPB (106 U/mg DCW and 85 U/mg DCW, respectively). These activities were slightly lower than those observed by Laszlo et al., with immobilized CALB in various inorganic supports [35]. That indicates a good hydrolytic activity of the whole cell catalysts towards triglycerides.

**Fig 3:**
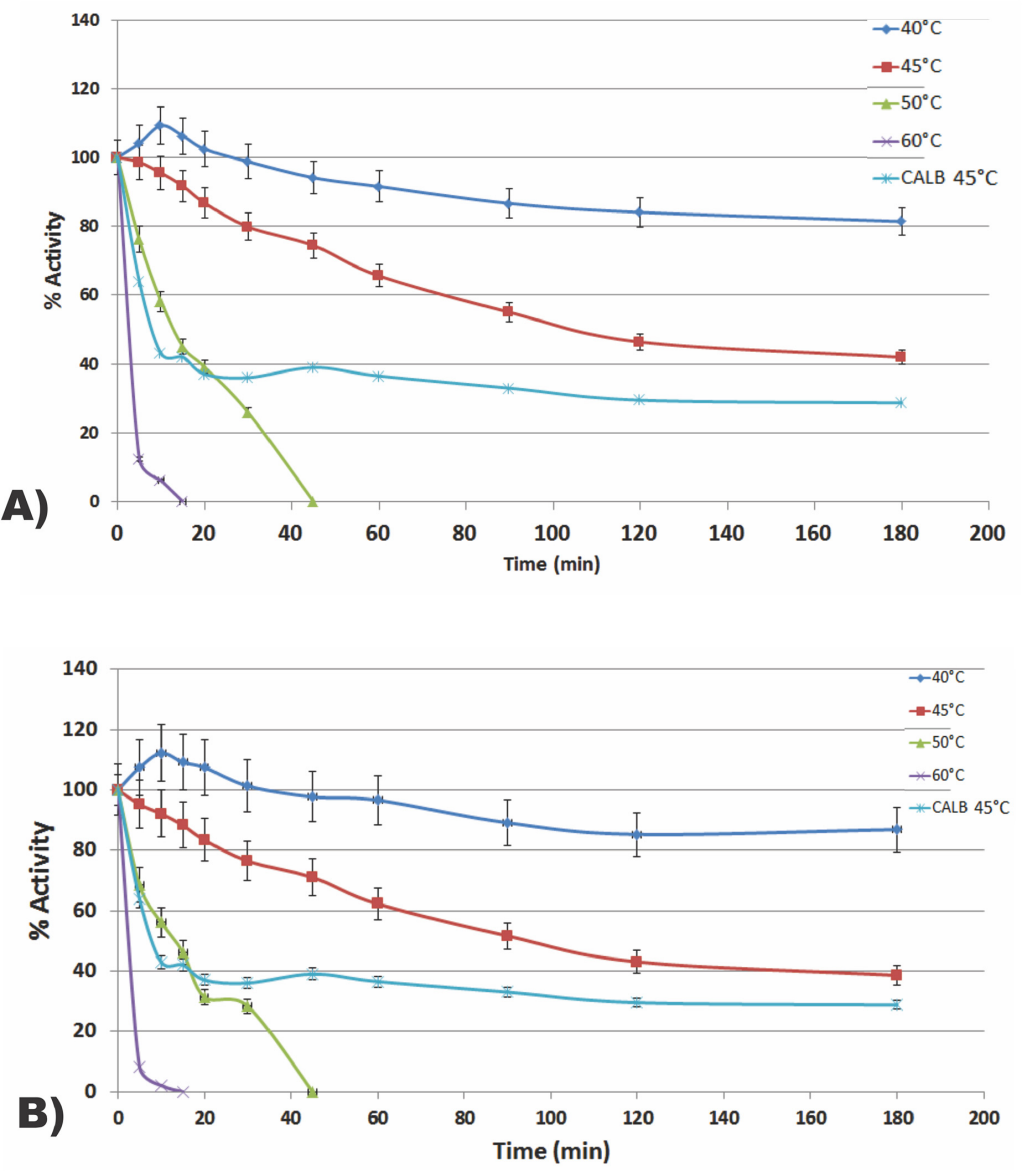
Temperature stability of PIRLIPB (A) and FLO9LIPB (B). In the blue temperature of 40°C, red 45°C, green 50°C and 60°C Purple. In light blue, Lipozyme CALB at 45°C.The y-axis represent the percentage of remaining activity after incubation in the each temperature.

Since many of the reactions catalyzed by lipases benefit from higher temperatures, thermostability is one of the most important features in the field of biocatalysis [1,2]. Fig 3 show the thermostability of the WCCs in the range of 40°C to 60°C.

Again, the catalysts behaved similarly showing conservation of ~85% of its activity after 3 hours incubation at optimum temperature of 40°C, and 40% at 45°C. At higher temperatures, there was denaturation of the enzyme. When compared to the free form of the lipase we observed an improvement of the stability of the immobilized enzymes: the free enzyme lost 60% of its activity in the first 10 minutes at 45°C. This shows that anchoring prompted to protect the lipase from thermal denaturation in both constructions. These results are similar to those reported by Kato *et al*. [34] who expressed a modified LipB anchored to Flo1 and noted a remaining 85% activity when incubated for 30 minutes at 40°C. However, this construction was more stable at higher temperatures, yielding approximately 50% of its activity after incubation at 60°C.

**Fig 4:**
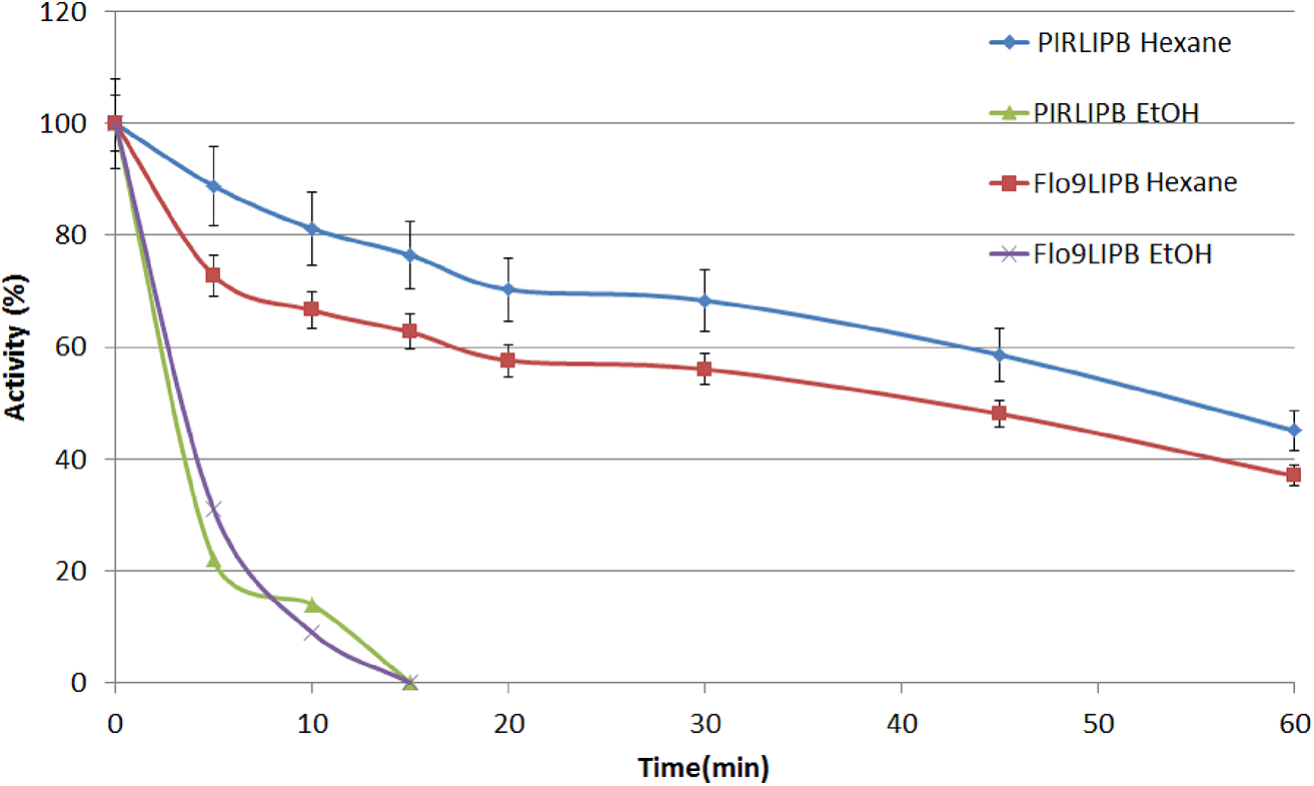
Protein stability in organic solvents. Blue line: PIRLIPB in hexane; red line: FLO9LIPB in hexane; green line: PIRLIPB in ethanol; purple line: FLO9LIPB in ethanol.

The stability of the catalyst towards to organic solvents was also tested. Lipases are known to catalyze numerous reactions of technological interest that take place in organic media. Stability towards organic solvents, thus becomes an important characteristic for biocatalysts. Fig 4 shows the performance of the constructions in ethanol and hexane.

The stabilities show the poor performance of the catalysts in ethanol, a polar solvent. Other more polar solvents were tested, such as methanol and acetonitrile, but the stability in high concentrations of these solvents was very low, with the catalyst losing activity after 5 minutes of incubation (data not shown). These results are in agreement with the literature—it is described that high concentrations of ethanol and other hydrophilic solvents can rapidly inactivate the enzyme [23, 36–38]. However, in hexane, the catalysts show significant results, maintaining 45% (PIRLIPB) and 36% (FLO9LIPB) of their activities after 1 hour incubation. This time it was observed significant differences between the stability of the anchors, the Pir1 construct was more stable in hexane. It has been previously reported that the biodiesel conversion reactions are carried out with controlled addition of methanol and ethanol to prevent denaturing of the YSD catalyst [36]. These same reactions are performed in organic solvents such as hexane and heptane, where these biocatalysts have been reported to stabilize and prevent denaturation of the enzyme. Jin *et al*. [23] studied the use of YSD cells for the production of biodiesel, using heptane as solvent. These authors also further investigated the use of co-solvents to increase the reaction yield. They reported that a large addition of methanol causes losses in yield. Using stepwise addition of the alcohols, the catalysts remained stable to organic solvents used in the reaction.

## Conclusion

The strategy of gene prospection used in this study resulted in the identification of an anchor (Flo9) not previously reported in the literature which is capable of being used for YSD. Both Flo9 and Pir were successfully used to display LipB, which is, to our knowledge the first report of the use of these anchors to immobilize a lipase,. Displayed LipB was hydrolytic active and showed thermostability, indicating that the anchors did protect the enzyme from hazardous conditions. The whole cell approach was able to increase the stability of the enzyme while maintaining the main biochemical characteristics of LipB. The catalysts were able to maintain more than 80% of its stability after 3 h incubation at 40°C also showing stability in organic solvents. This indicates that these cost-effective catalysts can be further used in various applications.
